# Biomarkers of Progression Independent of Relapse Activity—Can We Actually Measure It Yet?

**DOI:** 10.3390/ijms26104704

**Published:** 2025-05-14

**Authors:** Gabriel Bsteh, Assunta Dal-Bianco, Nik Krajnc, Thomas Berger

**Affiliations:** 1 Department of Neurology, Medical University of Vienna, 1090 Wien, Austria; gabriel.bsteh@meduniwien.ac.at (G.B.); assunta.dal-bianco@meduniwien.ac.at (A.D.-B.); nik.krajnc@meduniwien.ac.at (N.K.); 2 Comprehensive Center for Clinical Neurosciences and Mental Health, Medical University of Vienna, 1090 Wien, Austria

**Keywords:** multiple sclerosis, progression independent of relapse activity, measure, biomarker

## Abstract

Progression independent of relapse activity (PIRA) is increasingly recognized as a key driver of disability in multiple sclerosis (MS). However, the concept of PIRA remains elusive, with uncertainty surrounding its definition, underlying mechanisms, and methods of quantification. This review examines the current landscape of biomarkers used to predict and measure PIRA, focusing on clinical, imaging, and body fluid biomarkers. Clinical disability scores such as the Expanded Disability Status Scale (EDSS) are widely used, but may lack sensitivity in capturing subtle relapse-independent progression. Imaging biomarkers, including MRI-derived metrics (brain and spinal cord volume loss, chronic active lesions) and optical coherence tomography (OCT) parameters (retinal nerve fiber layer and ganglion cell-inner plexiform layer thinning), offer valuable insights, but often reflect both inflammatory and neurodegenerative processes. Body fluid biomarkers, such as neurofilament light chain (NfL) and glial fibrillary acidic protein (GFAP), are promising indicators of axonal damage and glial activation, but their specificity for PIRA remains limited. This review emphasizes the distinction between predicting PIRA—identifying individuals at risk of future progression—and measuring ongoing PIRA-related disability in real time. We highlight the limitations of current biomarkers in differentiating PIRA from relapse-associated activity and call for a clearer conceptual framework to guide future research. Advancing the precision and utility of PIRA biomarkers will require multimodal approaches, longitudinal studies, and standardized protocols to enable their clinical integration and to improve personalized MS management.

## 1. Introduction

Multiple sclerosis (MS) is traditionally characterized by inflammatory relapses and neurodegeneration-related disease progression [1]. However, increasing evidence suggests that progression independent of relapse activity (PIRA) represents a distinct pathophysiological process, challenging the conventional dichotomy between relapsing and progressive MS [2,3,4]. Originally, PIRA was defined as irreversible disability accumulation, as measured using the Expanded Disability Status Scale (EDSS), occurring in the absence of contemporary clinical relapses, as a demarcation from relapse-associated worsening (RAW), and accounting for more than 50% of disability accrual events and occurring in about 5% of people with MS (pwMS) per year [5]. However, when the definition is further refined to exclude cases with overt radiological markers of acute inflammation—such as contrast-enhancing lesions (CEL) or new/enlarging T2 lesions—this rate is significantly reduced. This stricter criterion has been termed disability progression independent of relapse and MRI activity (PIRMA) [5].

A fundamental question remains: Does PIRA reflect a purely non-inflammatory neurodegenerative process, is it still driven by residual, compartmentalized inflammation, or does it serve as an umbrella term encompassing both mechanisms?

A concept often mentioned in relation to PIRA is “smoldering MS”; the two terms are often used interchangeably, but they are not synonymous.

PIRA is a clinical term, while smoldering MS arises from neuropathological observations of slowly expanding chronic active lesions, ongoing axonal degeneration, diffuse meningeal and parenchymal inflammation, and widespread microglial activation beyond focal demyelinated plaques [6]. These processes contribute to disability accumulation beyond acute relapses and may underlie PIRA, but PIRA as a clinical entity does not necessarily equate to smoldering MS.

Given the heterogeneity of the underlying mechanisms, different biomarkers are likely needed to capture various aspects of PIRA, whether driven by neurodegeneration, chronic inflammation, or other yet undefined disease processes.

Distinguishing between biomarkers that predict PIRA and those that measure ongoing PIRA is essential. Predictive biomarkers can help identify pwMS at risk of future disability accrual, while measuring biomarkers should accurately quantify ongoing neurodegeneration independent of relapses.

This review critically evaluates current and emerging biomarkers in terms of their pathobiological correlates and their utility in predicting vs. measuring PIRA. Additionally, it examines the conceptual framework of PIRA and explores the need for further refinement to enhance its clinical and research applications.

## 2. Clinical Biomarkers

### 2.1. Expanded Disability Status Scale

The EDSS, a widely utilized tool for assessing neurological impairment in MS, evaluates disability across eight functional systems on a scale from 0 to 10 [7,8]. While it is applicable to all pwMS regardless of age or disease course, it has considerable methodological limitations. Studies have demonstrated that EDSS worsening and improvement occur at comparable rates in both relapsing and progressive MS, with the EDSS exhibiting low sensitivity to true disease progression, particularly in its lower range [9,10]. Although irreversible disability accumulation can arise at any disease stage through two distinct mechanisms—RAW and PIRA—the EDSS lacks the precision to reliably differentiate these pathways [11,12]. Also, there is some inter-rater variability strongly depending on the level of training of raters [13,14]. Moreover, the EDSS is disproportionately weighted toward ambulatory function, largely overlooking upper-extremity impairment and cognitive decline, and displays a non-linear change pattern over time [15]. Disability progression definitions based on the EDSS vary in key methodological aspects, including minimum required change, the use of a fixed vs. a roving baseline, and whether a change in EDSS must be confirmed over a specific period or sustained throughout the entire observation period [16,17]. A study in primary progressive MS showed progression rates ranging from 28% to 82%, with times to progression varying from 2653 to 550 days, driven primarily by the required minimal EDSS increase (accounting for up to a 24% difference in event rate), the confirmation period (up to 20%), and the choice of a roving vs. a fixed baseline (up to 12%) [18]. Another recent study demonstrated that pwMS experiencing PIRA after a first demyelinating event have an unfavorable long-term prognosis, particularly if PIRA occurs within the first five years of the disease, underscoring its predictive value [19]. Still, the EDSS appears to be more effective in measuring rather than predicting PIRA, highlighting its key limitation: its sensitivity is confined to detecting irreversible neuroaxonal damage post hoc while failing to capture future—potentially preventable—worsening.

### 2.2. Multiple Sclerosis Functional Composite

The Multiple Sclerosis Functional Composite (MSFC) is a composite score assessing short-distance ambulation via the timed 25-foot walk test (T25FW), upper-extremity function using the 9-hole peg test (9HPT), and cognitive performance assessed by the Paced Auditory Serial Addition Test (PASAT). Compared to the EDSS, the T25FW and 9HPT demonstrate greater sensitivity in detecting disability progression, with a clinically significant threshold for change defined as ≥20% [20,21,22]. However, the PASAT has shown limited sensitivity to subtle cognitive decline and is often poorly tolerated by pwMS due to its complexity and high cognitive demand [23]. Both 9HPT and PASAT are also subject to practice effects, reducing their reliability over repeated administrations. Furthermore, the EDSS plus composite score, which integrates the EDSS, T25FW, and 9HPT, has demonstrated approximately twice the sensitivity of the EDSS alone in detecting disability progression (59.5% vs. 24.7%, respectively). However, the inclusion of the 9HPT results in only a marginal increase in the detection of worsening events [24]. While a combination of measures offers greater sensitivity for detecting PIRA, consolidating them into a single metric may inadvertently increase measurement noise and decrease specificity [25,26]. Within PASAT components, the T25FW appears as the most robust and reliable clinical measure for detecting disease progression [27]. It exhibits good to excellent test–retest reliability, minimal inter-rater variability, and is easily applicable in real-world settings due to its brief administration time, with results attainable by trained staff [26].

### 2.3. Symbol Digit Modalities Test

The Symbol Digit Modalities Test (SDMT) is a valuable tool for assessing sustained attention, concentration, and visuomotor speed, which are often impaired in pwMS, but are not captured when relying solely on the EDSS [28,29]. In comparison to the PASAT, the SDMT demonstrates superior performance, with higher sensitivity and moderate specificity (91% and 60%, respectively), where a 4-point or 10% change is considered clinically relevant [30,31,32]. Notably, it correlates less strongly with the EDSS and other performance metrics (T25FW, 9HPT), providing supplementary insights into cognitive functions that are not captured by these measures [33]. Recently, approximately one-fifth of PIRA cases were attributed to isolated cognitive decline, as measured with the SDMT [34]. While the SDMT enhances sensitivity in detecting PIRA, its specificity is limited by the influence of overlapping comorbidities, such as depression and fatigue [35,36]. Additionally, the SDMT is susceptible to significant practice effects, particularly when administered at brief intervals, although these effects can be mitigated by changing scoring keys and diminish with disease progression [37,38].

An inherent but fundamental limitation of all clinical measures is their inability to detect subclinical changes before they manifest as measurable disability, which is predominantly linked to irreversible neuroaxonal damage and subsequent permanent impairment (Table 1). Moreover, most measures exhibit floor and ceiling effects, limiting their sensitivity to PIRA at the extremes, where pwMS may be either too mildly affected or too severely disabled to meet the required thresholds for documented disability progression.

## 3. Imaging Biomarkers

### 3.1. MRI Based Biomarkers

Brain and spinal cord atrophy; focal and diffuse chronic inflammation in the white and gray matter; and compartmentalized inflammation in the meninges are contributing imaging biomarkers for PIRA in MS (Table 1). Together, these imaging markers aim to uncover gradual ongoing neurodegeneration and to indicate the amount of repair failure, both ideally already in the early phase of the disease

#### 3.1.1. Both Brain and Spinal Cord Volume Loss

Brain and spinal cord volume loss are established markers of irreversible neurodegeneration in MS and also appear as strong predictors of further disability progression [39,40]. Recent studies have shown that pwMS with PIRA exhibit significantly greater brain volume loss than clinically stable pwMS—primarily due to cortical gray matter (GM) atrophy—along with increased spinal cord atrophy [41,42]. Additionally, the baseline volumes of the cervical cord, whole brain, and cerebral cortex have been identified as predictors of time to PIRA [42]. Moreover, thalamic volume loss has been shown to be an early and particularly sensitive and consistent marker of neurodegeneration across different MS phenotypes, which is also associated with progressive disability worsening in MS [43,44,45].

Nevertheless, volume measurement is challenged by certain technical factors of MRI such as scanner variability, acquisition protocols, segmentation techniques, and lesion filling, as well as biological influences like physiological aging and causes of pseudoatrophy (e.g., hydration, hormones, drugs) [46,47,48,49]. While a loss of 0.4% brain volume per year has been proposed as the pathological cut-off for whole brain volume loss in MS, it needs to be considered that even healthy adults display up to 0.3% annual volume loss, wherefore the observed effect sizes are often well within the margin of error due to technical and biological variabilities [50,51]. Although a variety of imaging postprocessing techniques like SIENA (Structural Image Evaluation, using Normalization, of Atrophy), SIENAX (Structural Image Evaluation, using Normalization, of Atrophy—Cross-sectional) or ANTs (Advanced Normalization Tools), as well as various deep-learning approaches, aim to improve volume measurement accuracy, these issues remain, particularly regarding scanner variability [48,52,53]. Thus, to overcome the challenge of distinguishing true atrophy from scanner noise, reliable assessment in individual pwMS is considered feasible only over observation periods of at least five years [46]. Nevertheless, measuring brain and spinal cord volume provides direct and objective real-time data on neurodegeneration.

However, the prediction of future volume loss, which would potentially have greater value for therapeutic interventions, is even more challenging. Due to temporal variability in volume loss rates (e.g., due to changing disease activity, treatment effects), reliance on a single MRI scan or short observation periods, as well as lack of standardized prediction models, future neurodegeneration is prone to both under- and overestimation [49]. Despite these issues, standardized, repeated volumetric measurements remain a main pillar for tracking MS progression. Advances in MRI consistency and AI-driven analysis tailored to MS will help overcome current technical and patient-related limitations, further strengthening future prediction models.

#### 3.1.2. Focal Neuroinflammation

*Chronic MS lesions* refer to long-standing areas of ongoing demyelination and neuroaxonal damage in the central nervous system, typically persisting for months or years. In MRI, these lesions typically show T2/FLAIR hyperintensity (1–2 mm isotropic) and often black holes on T1-weighted imaging (1 mm isotropic). However, they can be further categorized based on the presence of a paramagnetic rim and whether they expand over time, with differing impacts on PIRA.

*Paramagnetic Rim Lesions (PRLs)* are neuropathologically defined chronic active, slowly expanding lesions characterized by a pro-inflammatory rim of iron-laden microglia and macrophages surrounding the lesion border, leading to persistent tissue damage through demyelination, axonal degeneration, and remyelination failure, not only within the lesion, but also in the surrounding perilesional area [54,55]. PRLs are visualizable using phase-sensitive MRI sequences at 1.5, 3, and 7 T such as Quantitative Susceptibility Mapping (QSM; 0.8–1.4 mm isotropic), Susceptibility-Weighted Imaging (SWI), Magnitude, Phase, T2* (gradient echo T2-weighted imaging*), or R2* (1/T2*) (all 1–2 mm isotropic) [56,57]. Various studies have shown that PRLs are associated PIRA and appear as a key driver of more severe clinical disability [58,59]. A recent study showed that the appearance of new PRLs correlates with increased PIRA, while PRL disappearance is linked to reduced progression, highlighting their role as an imaging biomarker for potentially reversible pathology in contrast to atrophy as an irreversible biomarker for neurodegeneration [58]. Furthermore, PRLs were shown as predictors of future disability progression as pwMS with a higher burden of PRLs display a greater risk of suffering both PIRA and overall disability accrual [42,60].

*Slowly Expanding Lesions (SELs)* are another type of chronic active lesions characterized by their gradual expansion over time, but without the iron-driven pro-inflammatory rim [61]. SELs are detected using longitudinal deformation analysis based on conventional T1 and T2- weighted MRI-sequences by a Jacobian Algorithm calculating deformation fields between serial scans and identifying concentric lesion expansion over 1–2 years [61,62]. Therefore, detecting SELs necessitates a minimum of two MRI scans conducted over a period of at least one year. SELs have been associated with PIRA and are thought to drive neurodegeneration through persistent low-burning inflammation and progressive tissue damage [63].

The combined presence of PRLs and SELs is associated with even more severe disability accumulation as well as higher serum levels of neurofilament light chains (NfL) and glial fibrillaric acidic protein (GFAP), markers of axonal damage and astrocytic activation, further supporting their role in ongoing neuroaxonal damage and disease progression [64,65,66].

While *Non-Expanding Rim Lesions* do not enlarge, iron deposition may still exert a moderate neurotoxic effect, although their contribution to PIRA is lower than that of expanding PRLs and SELs. *Stable or even shrinking Non-Rim Lesions* reflect gliosis rather than ongoing inflammation, with, therefore, only a low impact on PIRA [55]. Together, PRLs and SELs are robustly associated with concurrent PIRA and appear as strong predictors of future PIRA as well [42,59,67]. However, detection of PRLs and SELs requires advanced phase-sensitive imaging (SWI, QSM), which is technically demanding and not widely accessible. Moreover, manual identification is time-intensive and inconsistent, while automated methods still need validation and longitudinal tracking is costly and resource-intensive, limiting feasibility in routine care [68]. Additionally, identifying lesions at risk of evolving into PRLs or SELs still remains uncertain, as underlying processes are not yet fully understood [69]. Furthermore, both PRLs and SELs have yet to be studied within the multimodal framework of established markers of disability accrual [19,42,70,71,72]. Addressing these challenges requires further research, standardization, and technological advancements.

*Cortical lesions (CL) and meningeal inflammation* are both thought to significantly contribute to PIRA. CLs are associated with gray matter atrophy, accelerated cortical thinning and neurodegeneration and are topographically associated with lymphoid aggregates in the meninges [73,74]. The presence of CLs is associated with more severe clinical disability accrual overall and higher rates of PIRA [41,75,76]. CLs can be detected best using high-field 7 T MRI with its high spatial resolution, but can also be visualized by advanced MRI techniques such as Phase-Sensitive Inversion Recovery (PSIR, 1–2 mm isotropic), Double Inversion Recovery (DIR, 1–2 mm isotropic) or a combination of MP2RAGE (0.8–1 mm isotropic) and T2* (1–2 mm isotropic) [74,77,78,79,80,81,82,83]. The most effective imaging modalities for detecting meningeal inflammation include 7 T MRI with delayed post-contrast FLAIR (0.8–1 mm isotropic), 3 T Real Inversion Recovery (Real-IR) MRI (1–2 mm isotropic), and 3 T delayed post-contrast FLAIR (1–2 mm isotropic) [84,85,86,87,88]. Though 7 T MRI provides the highest sensitivity for leptomeningeal enhancement, especially with delayed gadolinium imaging, 3 T delayed post-contrast FLAIR remains a valuable, more accessible tool in clinical settings. Still, these techniques are currently restricted by their limited availability, requirement of specific rater expertise and the lack of validated automated [68].

#### 3.1.3. Diffuse Neuroinflammation

Diffuse neuroinflammation in normal-appearing white matter (NAWM) has become a critical focus in understanding disease progression. This process is driven by the innate immune system operating behind an intact blood–brain barrier and occurs independently of peripheral immune cell infiltration, as NAWM displays significantly more distinct microglial activation compared to healthy controls [89]. Both focal and diffuse microglial activation can be visualized using PET imaging (2–3 mm isotropic), particularly with radioligands targeting the 18 kDa translocator protein (TSPO) [90,91,92]. Elevated TSPO uptake is associated with sustained neuroinflammation, contributing to PIRA with higher rates of lesion enlargement, brain volume loss, and higher sNfL levels, predicting a more severe disease trajectory with pre-lesional pathology [93,94]. A recent study has demonstrated significantly higher levels of white matter inflammatory activity by means of TSPO-PET in pwMS displaying PRLs, leading to larger lesions and faster disability progression [95]. Nevertheless, TSPO-PET imaging remains controversial because TSPO is expressed by various cell types—including pro-inflammatory and homeostatic microglia, astrocytes, and macrophages—which makes it challenging to interpret PET signals as a precise measure of microglial activation states [96]. On the other hand, TSPO-PET has proven valuable for in vivo phenotyping MS lesions, enabling differentiation between active, rim-active, and inactive lesions [97]. In essence, this underscores the need for cautious interpretation. Emerging PET tracers more specifically targeting microglial markers, such as the macrophage colony-stimulating factor 1 receptor (CSF1R), show promise in providing better insights into microglial dynamics and their role in neuroinflammation [98]. These advancements in PET imaging are crucial for developing and monitoring the innate immune system—and new therapeutic strategies aimed at modulating chronic microglial activity—to improve understanding and potentially mitigate PIRA [99].

While PET imaging provides insights into the cellular and molecular mechanisms underlying neuroinflammation, advanced MRI techniques complement these findings by capturing the structural and metabolic consequences of diffuse inflammation. For instance, magnetic resonance spectroscopy (MRS) studies (3–4 mm isotropic) have demonstrated that metabolic alterations in normal-appearing white matter (NAWM)—such as an elevated myo-inositol to total creatine (tCr) ratio and reduced N-acetylaspartate (NAA) to tCr ratio—correlate with increased physical disability [100,101,102]. Similarly, diffusion tensor imaging (DTI; 2 mm isotropic) studies report that lower fractional anisotropy (FA) and higher mean diffusivity (MD) reflect microstructural damage associated with reduced performance on the T25FW and increased EDSS scores [103,104]. Moreover, neurite orientation dispersion and density imaging (NODDI; 2 mm isotropic) studies have indicated that an increased orientation dispersion index (ODI) and reduced neurite density index (NDI) are linked to cognitive decline and disability [105,106]. Additionally, myelin water imaging (MWI; 1–2 mm isotropic) findings show that the myelin water fraction (MWF) in NAWM is more reduced in progressive patients compared to those with relapsing-remitting MS [107]. Finally, magnetization transfer imaging (MTI; 1–2 mm isotropic) measures—sensitive to both myelin density and microglial activation—have revealed NAWM abnormalities indicative of reduced myelin density [108,109,110]. Detectable lesion diameters for MRS range from 3 to 20 mm, while DTI and NODDI as well as PET typically detect lesions between 2 and 20 mm [91,111,112]. All other above-mentioned modalities detect lesions with diameters ranging from 1 to 20 mm [113,114,115,116,117]. Lesions larger than 20 mm are relatively rare in MS; most MS lesions are smaller than 10 mm in diameter. This typical size range is clinically significant, as it encompasses the majority of lesions relevant for monitoring disease progression and evaluating treatment response [118]. Together, PET and MRI may provide synergistic and complementary insights into the inflammatory, metabolic, and microstructural changes underlying PIRA.

### 3.2. Optical Coherence Tomography

Optical coherence tomography (OCT) is a non-invasive, accessible imaging technique that uses near-infrared light to produce high-resolution images of the retina [119]. It allows for precise measurement of the peripapillary retinal nerve fiber layer (pRNFL) and ganglion cell-inner plexiform layer (GCIPL), which in turn serve as reliable biomarkers for neuroaxonal degeneration in MS [120]. With the advent of spectral domain OCT (SD-OCT) providing automated segmentation algorithms and advancements through eye tracking and averaging multiple images, validity has improved to the extent that even small micrometer-scale changes can be reliably reproduced [121,122,123,124].

#### 3.2.1. Inner Retinal Layer Thickness

Several studies have shown that reduced pRNFL and GCIPL thicknesses in eyes without a history of optic neuritis (ON) are associated with a higher risk of future disability progression. Stratifying pwMS by retinal thickness indicates that those with the lowest tier of pRNFL and GCIPL thickness are at a 2- to 4-fold increased risk of their disability worsening within subsequent years [125,126,127,128]. The likely pathophysiological explanation for this association is that pwMS with lower retinal thickness have already accumulated more pronounced neuroaxonal damage and thus have less neuroaxonal reserve, thereby decreasing their capacity for compensation and increasing their vulnerability to further disability accrual.

#### 3.2.2. Inner Retinal Layer Thinning

The pathophysiology of MS-associated progressive retinal atrophy independent of ON is not fully understood, but it is largely thought to stem from MS lesions in the visual pathway, causing retrograde axonal degeneration and/or trans-synaptic degeneration of retinal cells. Retinal thinning is fastest early in the disease process and tends to plateau as the disease progresses, likely reflecting a floor effect [129,130,131].

Inner retinal layer thinning in MS occurs gradually at mean rates of 1–2 µm/year for pRNFL and 0.6–1.8 µm/year for GCIPL, with increased thinning observed in association with PIRA and elevated sNfL levels, whereby these rates markedly exceed the age-related thinning in healthy individuals (0.2–0.5 µm/year for pRNFL and 0.1–0.3 µm/year for GCIPL) [132,133,134]. Increased velocity of retinal layer thinning is a strong predictor of future disability accrual [127]. Of note, GCIPL thinning significantly exceeds the strength of association found with pRNFL thinning, and is also superior to cross-sectionally measured retinal thicknesses [127]. Monitoring retinal layer thinning represents a compelling model for MS-associated neurodegeneration and, thus, a promising candidate biomarker for measuring PIRA.

Despite growing evidence supporting the use of OCT in MS management, comprehensive longitudinal studies are needed to validate its role in monitoring neurodegeneration. Retinal layer thinning, although informative, lacks specificity, as it can also occur in other neurodegenerative diseases and is influenced by confounders such as ethnicity and coexisting neurological or ophthalmological conditions [135,136]. Additionally, technical limitations persist, including the floor effect, which can be mitigated by using percentage rather than absolute measurements of retinal layer thinning. Furthermore, while retinal thinning is predictive of PIRA with statistical significance on a group level, the absolute effect size of OCT findings is small and therefore prone to be overshadowed by technical and/or biological variability [137]. Automatic segmentation also requires quality control, which is partially addressed by using OSCAR-IB criteria. And, last but not least, OCT availability currently remains limited in most MS centers worldwide. Although OCT has demonstrated utility not only in measuring PIRA but also in predicting future disability progression, its sensitivity in detecting subclinical relapse-independent progression in real-time remains low.

## 4. Body Fluid Biomarkers: Neurodegeneration vs. Inflammation

### 4.1. Neurofilament Light Chain

Neurofilament Light Chain (NfL), a structural protein of axonal cytoskeleton, which is released into the cerebrospinal fluid (CSF) and subsequently the peripheral blood following neuroaxonal injury, serves as a sensitive but unspecific marker of axonal damage [138]. Until recently, NfL studies were largely confined to CSF due to the insufficient sensitivity of early detection systems, which were unable to accurately quantify the physiologically lower levels of sNfL, thereby limiting its ability to be studied and its clinical applicability [139]. The advent of Single Molecule Array (Simoa^®^) technology has revolutionized this field by enabling highly sensitive quantification of NfL not only in CSF, but also in serum and plasma [140]. Notably, multiple studies have demonstrated a strong correlation between CSF and serum NfL (sNfL) levels, facilitating the use of sNfL as a readily accessible biomarker for serial monitoring [140].

Whilst sNfL is a well-established marker of anti-inflammatory treatment response in active MS, its capacity to predict disease progression, especially when acute inflammatory activity is suppressed by high-efficacy DMTs, remains limited [141,142,143]. Nonetheless, numerous studies have indicated that increased sNfL levels are associated with concomitant disability progression as well as future disability accrual—although to a lesser extent [144,145,146,147]. Despite its promise, several critical limitations of sNfL need to be addressed. sNfL levels are influenced by age, body mass index, and confounding factors, such as physical activity, trauma, and small vessel disease [141,148]. Previous studies have shown that annual sNfL measurements account for only about 15–20% of the variation in brain volume loss, indicating that a substantial proportion of neuroaxonal loss remains unreflected by sNfL levels alone [149]. Whilst higher sNfL levels are indeed associated with an increased risk of PIRA, they also rise with clinical and radiological activity, limiting their specificity for non-relapse-related events. Most of the evidence supporting its use comes from group-level analyses, raising concerns about its reliability in making individual clinical decisions.

### 4.2. Glial Fibrillary Acidic Protein

GFAP is an intermediate filament protein expressed in astrocytes whose upregulation plays a pivotal role in the formation of extended, thickened astrocytic processes that are characteristic of reactive astrogliosis at the site of the injury [150]. This process does not always result in glial scar formation, as its re- or demyelinating potential is influenced by various factors, including the timing of injury, the lesion microenvironment, and interactions with other cell types and signaling molecules that regulate astrocyte activation [151]. However, extensive astrocytosis can lead to the development of astroglial scarring, which is increasingly recognized as one of the key drivers of disease progression in MS [152].

Recent studies have suggested the superior accuracy of GFAP compared to sNfL in distinguishing PIRA, underscoring the role of compartmentalized inflammation within the CNS as a key driver of smoldering MS, with a combined measurement further enhancing diagnostic precision [152,153,154]. Notably, unlike sNfL, GFAP does not appear to increase during acute inflammation in the CSF or serum, nor is it influenced by DMTs [155]. Thus, the simultaneous assessment of GFAP and sNfL may provide valuable insights for differentiating RAW from PIRA, offering a more refined approach to monitoring MS disease course [147,152]. Moreover, GFAP has been shown to be valuable not only in capturing disability accrual, but also as a reliable prognostic biomarker for future PIRA, further underscoring its complementary role alongside sNfL [66,152]. However, serum GFAP levels exhibit only a low to moderate correlation with their CSF counterparts, potentially limiting their utility in routine clinical practice [156].

## 5. Conceptual Challenges in Defining and Quantifying PIRA—What Are We Measuring?

The definition of PIRA remains an evolving concept with the aim to detect “non-inflammatory” neurodegeneration in MS [157]. The original term PIRA is defined as disability accumulation occurring without contemporary clinically overt relapses [3,4].

**Figure 1:**
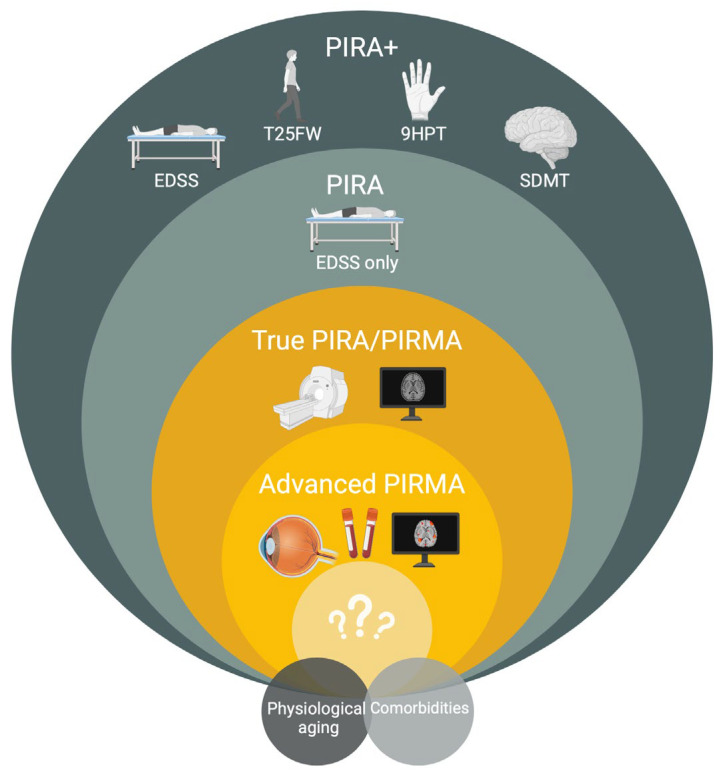
Peeling back the layers of PIRA. EDSS: Expanded Disability Status Scale. PIRA: progression independent of relapse activity. PIRMA: progression independent of relapse and magnetic resonance imaging activity. SDMT: Symbol Digit Modalities Test. T25FW: timed 25-foot walk test. 9HPT: 9-hole peg test.

On the one hand, this definition lacks specificity for “independency of inflammation”, as more than half of PIRA observed events occur in association with radiological evidence of acute inflammation [11]. Still, requiring the absence of contrast-enhancing or new T2 lesions—referred to as PIRMA or “true PIRA”—does not fully exclude compartmentalized or smoldering inflammation [5,157]. Adding additional dimensions using the biomarkers derived from advanced MRI techniques, (such as PRL, SEL, and cortical lesions), OCT, and serum biomarkers—referred to as advanced PIRMA—may further increase specificity to delineate neurodegeneration independent of inflammation [68] (Figure 1). However, implementing this approach poses significant challenges through the added complexity in clinical trials and even more in clinical routines.

Even if such a complex comprehensive assessment is applied and we find that disability worsening occurs in the absence of detectable inflammatory markers, we have to acknowledge that this is a clinical manifestation of underlying processes that we do not fully understand, and that we currently still lack the tools to depict relevant contributors to MS pathology underlying neurodegeneration independent of inflammation.

Moreover, distinguishing clinical worsening due to MS-associated pathobiology from changes associated with physiological aging and comorbidities remains a significant challenge. As pwMS age, factors such as vascular disease, reduced remyelination capacity, mitochondrial dysfunction, and overall frailty may contribute to functional decline. As the relative contributions of these mechanisms remain unclear, this further complicates the attribution of disability worsening or neurodegeneration solely to MS-related mechanisms in order to define PIRA as a discrete entity [158].

On the other hand, the sensitivity of detecting PIRA increases when broader measures of disability are incorporated beyond the EDSS, which primarily reflects motor disability and ambulation. More granular functional assessments, such as the T25FW, 9HPT, and SDMT, capture subtle changes in walking speed, upper limb function, and cognitive processing, respectively. Including these measures in PIRA definitions may allow for the earlier detection of disability accumulation that would otherwise go unnoticed using EDSS alone. Some studies have even proposed expanding the definition further to include patient-reported outcomes (PROs), aiming to capture subjective worsening of neurological function that may not yet be reflected in objective clinical testing [159]. However, whether this concept represents a refinement when weighing the trade-off between potentially increased sensitivity and likely decreased specificity of PIRA remains an open question.

The role of DMTs introduces an additional challenge: Should PIRA or any more refined definition be considered a form of treatment failure? Most DMTs primarily target relapse-associated disease activity, and while they may slow overall disability accrual, their efficacy in preventing PIRA is poorly studied and appears to differ only slightly between agents [5]. This raises a dilemma: If a patient on a high-efficacy DMT experiences PIRA, does this signify that the treatment is inadequate at addressing the underlying mechanisms of progression, or is PIRA simply an inevitable part of MS, independent of inflammatory suppression?

A major challenge in defining and quantifying PIRA is the long timeframe required to observe disability worsening with sufficient precision. Unlike relapses, which present acutely, PIRA-related worsening may occur gradually over months or years, making short-term assessments unreliable. Frequent high-resolution clinical follow-ups are essential to distinguish true progression from measurement variability or transient neurological fluctuations [5].

Rather than a strict dichotomy between relapse-associated worsening (RAW) and PIRA, MS progression likely exists on a continuum, influenced by a combination of acute inflammatory events, chronic inflammation, neurodegeneration, and aging. Some pwMS exhibit a mixed phenotype, where both relapse-dependent and relapse-independent disability accrual coexist, complicating classification. Moreover, the transition from relapsing-remitting MS (RRMS) to secondary progressive MS (SPMS) is gradual, raising the question of whether PIRA and SPMS represent distinct processes or different points on the same spectrum [160].

Despite these challenges, PIRA remains a useful concept because it highlights that many pwMS accumulate disability despite not suffering relapses. However, its current definition may be too broad to guide treatment decisions effectively. Given that differences between DMTs in preventing PIRA appear minimal, a deeper understanding of its underlying mechanisms is needed. Identifying biomarkers capable of differentiating inflammatory from neurodegenerative drivers will be crucial for refining the concept and determining whether PIRA represents treatment failure or an inevitable consequence of disease progression.

## 6. Future Directions for Biomarker Research

Multimodal comprehensive approaches with long observation and high granularity integrating clinical assessments, advanced imaging, and fluid biomarkers would contribute essentially to differentiate PIRA from other factors influencing disease course. Up to now, PIRA assessment remains limited to clinical trials, as its use in practice is highly controversial. Accurate detection would require standardized clinical exams including not only the EDSS, but also the T25FW and 9HPT, as well as SMDT or cognitive testing, as they can contribute to disability outside conventional relapse definitions. Furthermore, comprehensive MRI protocols and frequent visits beyond standard care with confirmation scores recorded ideally at 6 or 12 months. Expanding PIRA criteria to include cognitive decline, limb function, digital assessments, and patient-reported measures may further enhance sensitivity, but would make routine clinical application even more complex [68].

Advanced methodologies provide a comprehensive framework for dissecting the contributions of diverse pathogenic mechanisms to disability accrual, integrating insights from imaging modalities, fluid biomarkers, genetic variants, and proteomics. Any residual unexplained disability progression may offer pivotal insights, directing future research toward uncovering previously unrecognized drivers of neurodegeneration.

### 6.1. Multi-Omics and Systems Biology Approaches

Integrating proteomics, transcriptomics, and metabolomics holds significant potential for identifying novel biomarkers and elucidating the molecular mechanisms driving PIRA. Proteomics enables the identification of proteins and their post-translational modifications, while transcriptomics provides insight into gene expression changes associated with oxidative stress and mitochondrial dysfunction. Metabolomics detects alterations in glutamate, lactate, and lipid metabolism, which may serve as early indicators of neurodegeneration. The convergence of multi-omics approaches enhances the identification of key pathological pathways and could significantly improve the early detection of silent disease progression. Furthermore, the integration of AI-driven models with multi-omics data have the potential to revolutionize early intervention strategies and enable personalized treatment approaches in MS [161].

### 6.2. AI and Predictive Models

Machine learning models are poised to significantly enhance predictive accuracy by integrating clinical, imaging, and fluid biomarker data, aiding in the early identification of pwMS at higher risk of PIRA and facilitating personalized treatment strategies [161]. Combining these modalities enhances disease progression prediction, with some models already reporting a balanced accuracy of about 87%, highlighting its potential for clinical application [162]. Various research projects currently focus on leveraging AI for personalized prognostic modeling, optimizing treatment strategies, and improving patient outcomes. However, significant challenges remain, including data heterogeneity, model interpretability, and the necessity for standardized longitudinal data collection. Overcoming these obstacles requires close collaboration between clinicians, researchers, and data scientists to develop robust, transparent, and clinically applicable AI-driven models for MS management.

### 6.3. Clarifying the Definition of PIRA

Our evolving understanding of disease progression, coupled with the intricate interplay between inflammation and neurodegeneration, underscores the need to transition from a clinically based definition of PIRA to a more biologically driven framework [160]. Recent refinements, such as PIRMA, attempt to address this by excluding cases with new MRI lesions, thereby reducing reported PIRA events and highlighting the contribution of subclinical inflammation to progression. The overlap between RAW and PIRA, particularly when disability worsening follows initially overtly asymptomatic lesions, further complicates this distinction and raises the question of whether such lesions should be classified under RAW [5]. While implementing these conceptual shifts in clinical practice presents challenges, they align with recent recommendations advocating for MS classification based on disease-driving mechanisms rather than traditional clinical descriptors [160]. To enhance disease monitoring and optimize targeted MS therapies, refining PIRA definitions and integrating advanced imaging techniques is essential [68,160].

## 7. Conclusions

While clinical and paraclinical biomarkers hold promise for detecting PIRA, their utility remains limited due to the challenge of differentiating PIRA from other forms of disease activity and physiological aging. A fundamental issue is the lack of a precise definition of PIRA, which complicates the evaluation of existing biomarkers and their ability to accurately capture this phenomenon. Moving forward, refining the conceptual framework of PIRA will be essential, particularly by distinguishing predictive biomarkers from those that measure disease progression. Establishing clearer criteria for PIRA will not only enhance biomarker research, but will also facilitate their translation into clinical practice, ultimately improving patient care. Future studies should focus on standardizing definitions and methodologies to ensure that biomarkers effectively reflect underlying disease mechanisms and contribute meaningfully to both diagnosis and treatment strategies.

## Figures and Tables

**Table 1:** Markers of ongoing and predictors of future PIRA.

Modality	Measure	Marker of Ongoing PIRA	Predictor of Future PIRA
Clinical	EDSS	yes	Limited value
Clinical	T25FW	yes—within PIRA plus	Limited value
Clinical	9HPT	Marginal value within PIRA plus	Limited value
Clinical	SDMT	yes—within PIRA plus	Limited value
MRI	T2L	Negative marker within PIRMA	Limited value as negative predictor
MRI	Brain/spinal cord atrophy	yes	Potential predictor with limited evidence
MRI	PRL	yes	Potential predictor with limited evidence
MRI	SEL	yes	Potential predictor with limited evidence
MRI	CL/meningeal inflammation	Positive association	Potential predictor with limited evidence
PET-TSPO	Microglial activation	Positive association	Potential predictor with limited evidence
OCT	pRNFL	Negative association	Potential predictor with limited evidence
OCT	GCIPL	Negative association	Potential predictor with limited evidence
Fluid biomarkers	sNfL	Positive association but lacks specificity	Potential predictor but lacks specificity
Fluid biomarkers	GFAP	Positive association	Potential predictor with limited evidence

Abbreviations: CL: cortical lesion. DTI: diffusion tensor imaging. EDSS: Expanded Disability Status Scale. GFAP: glial fibrillary acidic protein. MRI: Magnetic Resonance Imaging. PIRA: progression independent of relapse activity. PRL: Paramagnetic Rim Lesion. SEL: Slowly Expanding Lesion. SDMT: Symbol Digit Modalities Test. sNfL: serum neurofilament light chain. T25FW: timed 25-foot walk test. 9HPT: 9-hole peg test. T2L: T2-weighted lesion. TSPO-PET: Translocator Protein Positron Emission Tomography.

## Data Availability

This review article contains no datasets generated or analyzed during the current study.
